# Optical coherence tomography features in vitreoretinal lymphoma compared with non-infectious uveitis

**DOI:** 10.1186/s12886-024-03513-7

**Published:** 2024-06-13

**Authors:** Lulwa El Zein, Wendy M. Smith, Launia J. White, David O. Hodge, Timothy W. Olsen, Jose S. Pulido, Lauren A. Dalvin

**Affiliations:** 1 Department of Ophthalmology, Mayo Clinic, 200 1st St SW, Rochester, MN 55905 USA; 2 Department of Quantitative Health Sciences, Jacksonville, FL USA; 3 Wills Eye hospital, Philadelphia, PA USA

**Keywords:** Vitreoretinal lymphoma, Uveitis, Sarcoidosis, Vitritis, Intermediate uveitis

## Abstract

**Background:**

Vitreoretinal lymphoma (VRL) is a rare intraocular malignancy that poses a diagnostic challenge due to the non-specific clinical presentation that resembles uveitis. The use of spectral domain optical coherence tomography (SD-OCT) has emerged as a valuable imaging tool to characterize VRL. Therefore, we sought to determine the specific OCT features in VRL compared to the uveitides.

**Methods:**

Retrospective chart review of patients who were seen at Mayo Clinic from January 1, 2010 through December 31, 2022.

The medical records and SD-OCT images at time of initial presentation were reviewed in patients with biopsy-proven VRL, intermediate uveitis, or biopsy-confirmed sarcoid posterior uveitis. Patients with VRL or similar uveitides including intermediate uveitis or sarcoid posterior uveitis were included.

**Results:**

There were 95 eyes of 56 patients in the VRL group and 86 eyes of 45 patients in the uveitis group, of whom 15 (33.3%) were diagnosed with intermediate uveitis and 30 (66.7%) with sarcoid chorioretinitis. The SD-OCT features more commonly seen at initial presentation in VRL patients (vs. uveitis) included preretinal deposits (31.6% vs. 9.3%, *p* = 0.002), intraretinal infiltrates (34% vs. 3.5%, *p* < 0.001), inner retinal hyperreflective spots (15.8% vs. 0%, *p* < 0.001), outer retinal atrophy (22.1% vs. 2.3%, *p* < 0.001), subretinal focal deposits (21.1% vs. 4.7%, *p* = 0.001), retinal pigmented epithelium (RPE) changes (49.5% vs. 3.5%, *p* < 0.001), and sub-RPE deposits (34.7% vs. 0%, *p* < 0.001). Features more frequently seen in uveitis included epiretinal membrane (ERM) (82.6% vs. 44.2%, *p* < 0.001), central macular thickening (95.3% vs. 51.6%, *p* < 0.001), cystoid macular edema (36% vs. 11.7%, *p* < 0.001), subretinal fluid (16.3% vs 6.4%, *p* = 0.04), and subfoveal fluid (16.3% vs. 3.2%, *p* = 0.003). Multivariate regression analysis controlling for age and sex showed absence of ERM (OR 0.14 [0.04,0.41], *p* < 0.001) and absence of central macular thickening (OR 0.03 [0,0.15], *p* = 0.02) were associated with VRL as opposed to uveitis.

**Conclusion:**

OCT features most predictive of VRL (vs. uveitis) included absence of ERM and central macular thickening.

## Introduction

Vitreoretinal lymphoma (VRL) is a rare intraocular malignancy that makes up less than 1% of all intraocular tumors [1]. Most VRL cases are non-Hodgkin, diffuse, large B-cell lymphoma [2]. Accurate diagnosis of VRL is especially important given that 60–70% of patients with primary VRL will eventually develop central nervous system (CNS) lymphoma that has a mortality rate of 70% at 33 months [2–4].

VRL is a great mimicker in ophthalmology. Diagnosis is challenging because the clinical presentation is variable and non-specific. Patients typically will have vitritis with or without retinal infiltrates that are difficult to distinguish from uveitis. Therefore, VRL can be easily mis-diagnosed, mistreated, and commonly result in delayed diagnosis and delayed appropriate treatment [5]. While a uveitis evaluation requires blood tests and various imaging studies to assess for systemic infectious and inflammatory causes, [6] physicians need to know when to suspect VRL and seek appropriate evidence for the presence of malignant lymphoma. When a diagnosis is uncertain, a biopsy is necessary to obtain a more definitive, diagnostic specimen from the aqueous, vitreous, choroid or retina; however, all biopsies may be associated with potential surgical risks and complications [7].

With advances in ophthalmic imaging, high resolution, detailed imaging features of VRL on spectral domain optical coherence tomography (SD-OCT) have been recently described [8–10]. These include cells in the posterior vitreous, neurosensory retinal hyperreflective foci, subretinal material, and reactive changes within the retinal pigment epithelium (RPE) [8, 9, 11, 12]. Direct comparisons of OCT features between VRL and non-infectious uveitides have been limited. To better understand the diagnostic value of OCT in VRL, we compared OCT findings at initial presentation in patients with biopsy-proven VRL to those with non-infectious uveitis.

## Methods

### Study design

This study is a single-institution, retrospective, comparative study of patients diagnosed with VRL versus non-infectious uveitis at Mayo Clinic (Rochester, MN) from January 1, 2010, through December 31, 2022. This study was performed in accordance with the guidelines of the Declaration of Helsinki and was granted approval by the institutional review board of Mayo Clinic and informed consent was obtained from all subjects. Patients with VRL, posterior uveitis, or intermediate uveitis were reviewed. The search was performed using an internal search engine with the free text search terms: ‘’vitreoretinal lymphoma,’’ ‘’intermediate uveitis,’’ ‘’pars planitis,’’ ‘’posterior uveitis,’’ and ‘’sarcoidosis.’’.

### Patient selection

For study inclusion in the VRL group, patients required either a biopsy-proven diagnosis of VRL from one or both eyes, or clinical features highly suggestive of VRL in the setting of a confirmed, positive CNS biopsy for lymphoma. With bilateral ocular involvement, a positive biopsy from one eye only, or a positive CNS biopsy was required to include both eyes in the study. In the uveitis group, a non-infectious uveitis diagnosis was confirmed by a uveitis specialist with supporting evidence such as biopsy, supportive laboratory testing, and a favorable response to medical treatment. SD-OCT was performed at initial presentation to Mayo Clinic Rochester. In our analysis, uveitis was chosen for comparison due to clinical and diagnostic confusion that is common with VRL. Since infectious uveitis is diagnosed with serologic testing, only non-infectious uveitis was included. White dot syndromes were excluded since such diagnoses have very specific clinical ophthalmic findings. Due to the similarities to VRL, sarcoid posterior uveitis and intermediate uveitis were selected for comparison. Intermediate uveitis was defined as the presence of vitreous inflammation (cell and haze) with retinovascular leakage on fluorescein angiography imaging and the absence of discrete chorioretinal inflammatory lesions. Testing for infectious causes and associated systemic inflammatory processes was negative, and the uveitis improved with anti-inflammatory treatment with follow up period of at least 1 year. All patients with sarcoid posterior uveitis had choroidal, retinal, or chorioretinal lesions, and the diagnosis of sarcoidosis was confirmed by tissue biopsy (lung or cutaneous).

### Data collection

**Fig. 1:**
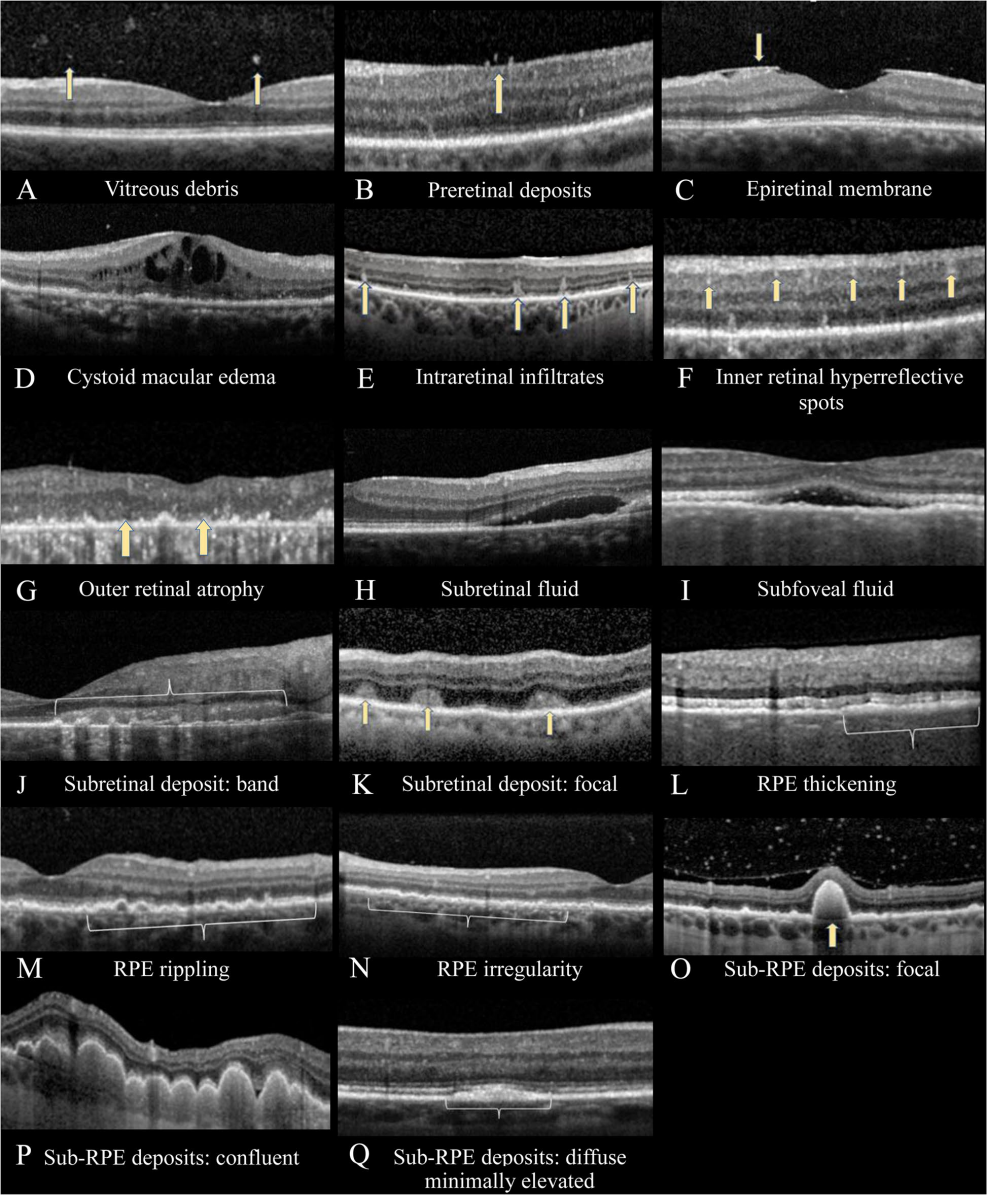
Spectral domain optical coherence tomography images displaying features of vitreoretinal lymphoma or uveitis. **A** Vitreous debris (arrow). **B** Preretinal deposits (arrows). **C** Epiretinal membrane (arrow). **D** Cystoid macular edema. **E** Intraretinal infiltrates (arrows). **F** Inner retinal hyperreflective spots (arrows). **G** Outer retinal atrophy (arrows). **H** Subretinal fluid. **I** Subfoveal fluid. **J** Subretinal deposit: band (bracket). **K** Subretinal deposit: focal (arrows). **L** Retinal pigment epithelium (RPE) thickening (bracket). **M** RPE rippling (bracket). **N** RPE irregularity (bracket). **O** Sub-RPE deposits: focal (arrow). **P** Sub-RPE deposits: confluent. **Q** Sub-RPE deposits: diffuse minimally elevated (bracket)

For all patients, demographic information (age, race, sex) and clinical history of diabetes mellitus and non-ocular cancer was collected. Ophthalmic data included bilateral or unilateral involvement, visual or ocular symptoms, duration of symptoms before diagnosis, and presenting visual acuity. OCT findings were assessed at the time of initial presentation and findings included posterior vitreous cells, preretinal deposits, ERM, retinal thickening, intraretinal fluid, intraretinal infiltrates, inner retinal hyperreflective spots, outer retinal atrophy, subretinal fluid, focal or band-like subretinal deposits, RPE thickening, rippling, irregularity, or atrophy, and focal, confluent or diffuse minimally elevated sub-RPE deposits (Fig. 1). Two authors (LAD, LEZ) independently assessed the OCT images of the patients. Any discrepancies in the identified OCT features were discussed between the authors.

### Statistical analysis

Continuous variables were summarized as mean ± SD and median (minimum, maximum) and compared between groups. Categorical variables were summarized as numbers and percentages. Generalized estimating equation (GEE) models were performed to assess for factors predictive of VRL vs. uveitis (unadjusted and adjusted by age and sex). Multivariate models were performed for dependent variable VRL vs. uveitis and included independent variables central macular thickening, epiretinal membrane, outer retinal atrophy, intraretinal infiltrates, subretinal deposits-dots, and RPE changes (unadjusted and adjusted by age and sex). Statistical analyses were performed using SAS (version 9.4; SAS Institute, Inc., Cary, North Carolina).

## Results

**Fig. 2:**
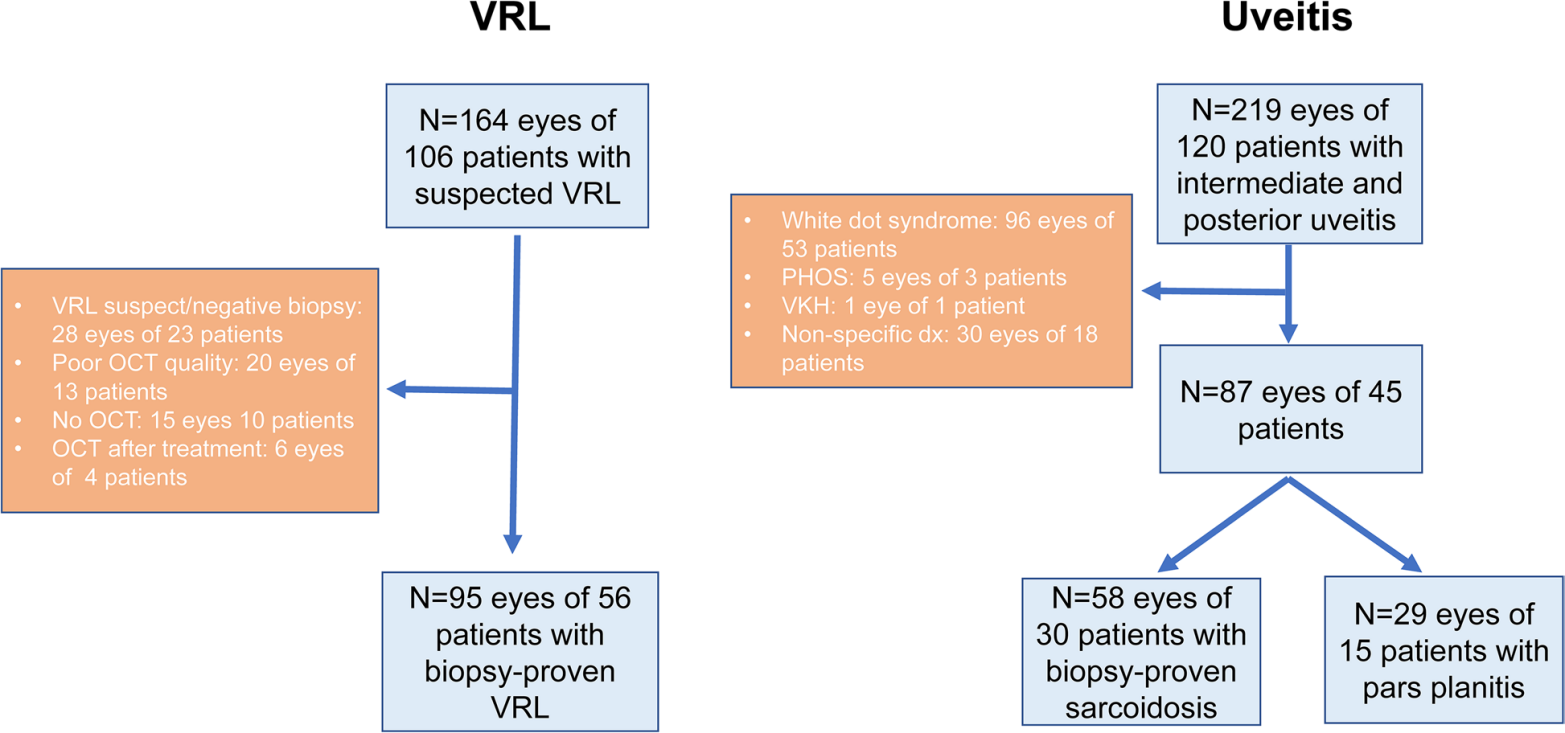
Patient selection flowchart

The search for VRL patients yielded 106 total patients with presumed VRL, of whom 56 (62.9%) had a positive tissue biopsy and were included in the study. There were 52 (93%) with ocular biopsy-proven disease (Supplemental table A). The remaining 4 patients (7%) had a positive CNS biopsy plus highly suggestive clinical features of VRL (Supplemental table B). Of 120 patients with a diagnosis of intermediate or posterior uveitis, a total of 45 patients (87 eyes) were included, of whom 15 (29 eyes) were diagnosed with undifferentiated intermediate uveitis and 30 (58 eyes) with sarcoid chorioretinitis (Fig. 2).

Table 1 describes patient demographics. VRL patients were older at initial presentation (67.9 vs. 43.6 years, *p* < 0.001). No significant differences were observed in sex, race, diabetes mellitus, or nonocular cancer history apart from CNS lymphoma, which was found in 29 patients (51.8%) in the VRL group.

**Table 1:** Optical coherence tomography features in vitreoretinal lymphoma compared with non-infectious uveitis: Demographics

	**VRL** **(*N*= 56)**	**Uveitis (*N*= 45)**	***p*-value**
**Age**			
Years, mean ± SD	67.9 ± 11.8	43.6 ± 19.6	** < 0.001**
median (range)	69.1 (29.0, 88.0)	47.0 (8.8, 74.1)	
**Sex, N (%)**			
Male	29 (51.8%)	25 (55.6%)	0.71
Female	27 (48.2%)	20 (44.4%)	
**Race, N (%)**			
White	50 (89.3%)	41 (91.1%)	0.13
African American	1 (1.8%)	2 (4.4%)	
Hispanic	3 (5.4%)	0 (0.0%)	
Asian	2 (3.6%)	0 (0.0%)	
Middle Eastern	0 (0.0%)	2 (4.4%)	
**Diabetes Mellitus, N (%)**			
Yes	7 (12.5%)	3 (6.7%)	0.33
No	49 (87.5%)	42 (93.3%)	

Bold values indicate significant *p*-value

*Abbreviations*: *N* Number of patients, *SD* Standard Deviation, *VRL* Vitreoretinal lymphoma

Clinical features at initial presentation are described in Table 2. The majority of VRL and uveitis cases were bilateral at presentation (76.8% for VRL vs. 93.3% for uveitis, *p* = 0.051). The mean duration of symptoms was shorter in the VRL group compared to the uveitis group (181.6 vs. 420.8 days, *p* = 0.09). Patients with VRL had worse presenting LogMAR visual acuity (0.37 [Snellen equivalent 20/46] vs. 0.25 [Snellen equivalent 20/35], *p* = 0.02). The VRL subjects were less likely to present with blurred vision (53.6% vs. 82.2%, *p* = 0.003) and more likely to report symptomatic floaters (60.7% vs. 37.8%,*p* = 0.022).

**Table 2:** Optical coherence tomography features in vitreoretinal lymphoma compared with non-infectious uveitis: Clinical features

	**VRL**	**Uveitis**	***p*-value**
**Disease Laterality, N (%)**	***N* = 56**	***N* = 45**	
Right	4 (7.1%)	2 (4.4%)	0.05
Left	9 (16.1%)	1 (2.2%)	
Both	43 (76.8%)	42 (93.3%)	
**Ocular Symptoms, N (%)**	***N* = 56**	***N* = 45**	
Blurred vision	30 (53.6%)	37 (82.2%)	**0.003**
Floaters	34 (60.7%)	17 (37.8%)	**0.02**
No symptoms	0 (0.0%)	1 (2.2%)	0.26
**Duration Symptoms, Days**	***N* = 32**	***N* = 33**	
Mean ± SD median (range)	181.6 ± 286.1 52 (2–1460)	420.8 ± 656.8 180 (2–3240)	0.09
**Best Corrected Visual Acuity in LogMAR**	***N* = 95 eyes**	***N* = 86 eyes**	
Mean ± SD median (range)	0.37 ± 0.58 0.18 (0.00–3.00)	0.25 ± 0.55 0.10 (0.00–4.00)	**0.02**

Bold values indicate significant *p*-value

*Abbreviations*: *N* Number of patients, *SD* Standard Deviation, *VRL* Vitreoretinal lymphoma

OCT features at initial presentation to the tertiary referral center is found in Table 3. Patients with VRL had significantly more frequent preretinal deposits (31.6% vs. 9.3%, *p* = 0.002), intraretinal infiltrates (34% vs. 3.5%, *p* < 0.001), inner retinal hyperreflective spots (15.8% vs. 0%, *p* < 0.001), outer retinal atrophy (22.1% vs. 2.3%, *p* < 0.001), subretinal deposits: focal (21.1% vs. 4.7%, *p* = 0.001), RPE thickening (13.7% vs. 0%, *p* < 0.001), RPE rippling (16.8% vs. 0%, *p* < 0.001), RPE irregularity (24.2% vs. 0%,* p* < 0.001), and sub-RPE deposits (focal [21.1% vs. 0%, *p* < 0.001], confluent [13.7% vs. 0%, *p* < 0.001], and diffuse minimally elevated [18.9% vs. 0%, *p* < 0.001]). Moreover, VRL patients were less likely to have an ERM (44.2% vs. 82.6%, *p* < 0.001), central macular thickening (51.6% vs. 95.3%, *p* < 0.001), intraretinal fluid (11.7% vs. 36%, *p* < 0.001), subretinal fluid (6.4% vs 16.3%, *p* = 0.04), or subfoveal fluid (3.2% vs.16.3%, *p* = 0.003) when compared to uveitis patients.

**Table 3:** Optical coherence tomography features in vitreoretinal lymphoma compared with non-infectious uveitis: OCT features

OCT features	VRL (*N* = 95)	Uveitis (*N* = 86)	*p-*value
**Vitreous features**			
Vitreous debris, N (%)	46 (48.4%)	47 (54.7%)	0.40
Preretinal deposits, N (%)	30 (31.6%)	8 (9.3%)	**0.002**
Epiretinal membrane, N (%)	42 (44.2%)	71 (82.6%)	** < 0.001**
**Retinal features**			
Central subfield thickness, Mean ± SD median (range)	286.6 ± 83.9 292.5 (151–771)	326.5 ± 70.9 313 (221–591)	** < 0.001**
Central macular thickening, N (%)	49 (51.6%)	82 (95.3%)	** < 0.001**
Cystoid macular edema, N (%)	11 (11.7%)	31 (36.0%)	** < 0.001**
Intraretinal infiltrates, N (%)	32 (34.0%)	3 (3.5%)	** < 0.001**
Inner retinal hyperreflective spots, N (%)	15 (15.8%)	0 (0.0%)	** < 0.001**
Outer retinal atrophy, N (%)	21 (22.1%)	2 (2.3%)	** < 0.001**
**Subretinal space**			
Subretinal fluid, N (%)	6 (6.4%)	14 (16.3%)	**0.04**
Subfoveal fluid, N (%)	3 (3.2%)	14 (16.3%)	**0.003**
Subretinal deposits-band, N (%)	17 (17.9%)	8 (9.3%)	0.09
Subretinal deposits-focal, N (%)	20 (21.1%)	4 (4.7%)	**0.001**
**RPE features**			
RPE changes, N (%)	47 (49.5%)	3 (3.5%)	** < 0.001**
RPE thickening, N (%)	13 (13.7%)	0 (0.0%)	** < 0.001**
RPE rippling, N (%)	16 (16.8%)	0 (0.0%)	** < 0.001**
RPE irregularity, N (%)	23 (24.2%)	0 (0.0%)	** < 0.001**
RPE atrophy, N (%)	8 (8.4%)	2 (2.3%)	0.07
Sub-RPE deposits, N (%)	33 (34.7%)	0 (0.0%)	** < 0.001**
Sub-RPE focal deposits, N (%)	20 (21.1%)	0 (0.0%)	** < 0.001**
Sub-RPE confluent deposits, N (%)	13 (13.7%)	0 (0.0%)	** < 0.001**
Sub-RPE diffuse minimally-elevated deposits, N (%)	18 (18.9%)	0 (0.0%)	** < 0.001**

Bold values indicate significant *p*-value

*Abbreviations*: *N* Number of eyes, *RPE* Retinal Pigmented Epithelium, *SD* Standard Deviation, *VRL* Vitreoretinal Lymphoma

Univariate GEE models were performed to assess for factors predictive of VRL (Table 4).Features associated with VRL on unadjusted analysis included preretinal deposits (odds ratio (OR) 4.5 [1.93–10.49], *p* = 0.01), intraretinal infiltrates (OR 14.28 [4.18–48.77], *p* < 0.001), outer retinal atrophy (OR 11.92 [2.7–52.55], *p* = 0.03), subretinal deposits-band and focal (OR 2.71 [1.28–5.74], *p* = 0.01), subretinal band deposits (OR 2.13 [0.87–5.21], *p* = 0.039), subretinal focal deposits (OR 5.47 [1.79–16.72], *p* = 0.003), and RPE changes (OR 27.09 [8.00–91.76],* p* < 0.001). Features less frequently observed in VRL that were more frequently seen with uveitis included an ERM (OR 0.17 [0.08,0.33], *p* = 0.012) and central macular thickening (OR 0.05 [0.02,0.15], *p* = 0.017). After adjusting for age and sex, features associated with VRL included intraretinal infiltrates (OR 8.67 [1.01,74.75],* p* = 0.05) and RPE changes (OR 16.95 [4.31,66.76], *p* = 0.002). Features associated with uveitis included an ERM (OR 0.17 [0.07,0.40], *p* = 0.002) and central macular thickening (OR 0.03 [0,0.20], *p* < 0.001).

**Table 4:** Optical coherence tomography features in vitreoretinal lymphoma compared with non-infectious uveitis: Univariate analysis for factors predictive of VRL unadjusted vs adjusted for age and sex

OCT features	Unadjusted		Adjusted by age and sex	
	**OR [95%CI]**	***p-*value**	**OR [95%CI]**	***p-*value**
**Vitreous features**				
Vitreous debris visible	0.78 [0.43–1.40]	0.71	1.19 [0.56, 2.56]	0.65
Preretinal deposits	4.50 [1.93, 10.49]	**0.01**	3.50 [0.82, 14.98]	0.09
Epiretinal membrane	0.17 [0.08, 0.33]	**0.01**	0.17 [0.07, 0.40]	**0.002**
**Retinal features**				
Central subfield thickness, (10-unit increase)	-0.08 [-0.13, -0.03]	0.06	-0.10 [-0.16, -0.04]	**0.03**
Central macular thickening	0.05 [0.02, 0.15]	**0.02**	0.03 [0.00, 0.20]	** < 0.001**
Cystoid macular edema	0.24 [0.11, 0.51]	0.14	0.18 [0.07, 0.48]	0.08
Intraretinal infiltrates	14.28 [4.18, 48.77]	** < 0.001**	8.67 [1.01, 74.75]	**0.05**
Inner retinal hyperreflective spots	N/A	** < 0.001**	N/A	N/A
Outer retinal atrophy	11.92 [2.70, 52.55]	**0.03**	22.45 [0.89, 568.94]	0.06
**Subretinal spaces**				
Subretinal fluid	0.35 [0.13, 0.96]	0.26	0.42 [0.13, 1.35]	0.22
Subfoveal fluid	0.17 [0.05, 0.62]	0.24	0.22 [0.05, 0.93]	0.13
Subretinal deposits-band and focal	2.71 [1.28, 5.74]	**0.01**	1.40 [0.40, 4.85]	0.60
Subretinal deposits-band	2.13 [0.87, 5.21]	**0.04**	1.05 [0.35, 2.62]	0.83
Subretinal deposits-focal	5.47 [1.79, 16.72]	**0.003**	6.03 [0.76, 48.10]	0.09
**RPE features**				
RPE changes	27.09 [8.00, 91.76]	** < 0.001**	16.95 [4.31, 66.76]	**0.002**
RPE thickening	N/A	** < 0.001**	N/A	N/A
RPE rippling	N/A	** < 0.001**	N/A	N/A
RPE Irregularity	N/A	** < 0.001**	N/A	N/A
RPE Atrophy	N/A	0.10	N/A	N/A
Sub-RPE deposits	N/A	** < 0.001**	N/A	N/A
Sub-RPE focal deposits	N/A	** < 0.001**	N/A	N/A
Sub-RPE confluent deposits	N/A	** < 0.001**	N/A	N/A
Sub-RPE diffuse minimally elevated deposits	N/A	** < 0.001**	N/A	N/A

Bold values indicate significant *p*-value

*Abbreviations*: *CI* Confidence Interval, *OR* Odd Ratio, *RPE* Retinal Pigmented Epithelium, *SD* Standard Deviation, *VRL* Vitreoretinal Lymphoma

Multivariate GEE models were performed (unadjusted and adjusted for age and sex) (Table 5) using statistically significant variables from the univariate analysis. Features that remained predictive of VRL included absence of central macular thickening (OR 0.03 [0.01,0.16], *p* = 0.04) and absence of ERM (OR 0.13 [0.04,0.40, *p* < 0.001). These features remained significant after adjustment for age and sex [(central macular thickening OR 0.03 [0,0.15], *p* = 0.02) and (ERM OR 0.14 [0.04,0.41], *p* < 0.001)]. Preretinal deposits and subretinal deposits-band were analyzed using separate multivariate regression analysis due to convergence issues when both variables were simultaneously considered (Supplemental Table C and D). Neither features was significant on multivariate modeling.

**Table 5:** Optical coherence tomography features in vitreoretinal lymphoma compared with non-infectious uveitis: Multivariate Cox regression analysis for risk of VRL unadjusted vs. adjusted for age and sex

	**Unadjusted**		**Adjusted by age and sex**	
	**OR [95%CI]**	***p-*value**	**OR [95%CI]**	***p-*value**
**OCT Features**				
Central macular thickening	0.03 [0.01, 0.16]	**0.04**	0.03 [0.00, 0.15]	**0.02**
Epiretinal membrane	0.13 [0.04, 0.40]	** < 0.001**	0.14 [0.04, 0.41]	** < 0.001**
Outer retinal atrophy	13.45 [0.39, 468.25]	0.13	12.32 [0.45, 336.33]	0.07
Intraretinal infiltrates	1.90 [0.30, 12.01]	0.32	1.69 [0.26, 10.76]	0.35
Subretinal deposits-focal	0.87 [0.19, 4.02]	0.50	0.89 [0.19, 4.28]	0.50
RPE changes	9.61 [1.55, 59.48]	0.53	10.26 [1.63, 64.65]	0.64
**Age (years)**	1.10 [1.05, 1.14]	** < 0.001**	NA	NA

Bold values indicate significant *p*-value

*Abbreviations*: *CI* Confidence Interval, *OR* Odd Ratio, *PED* Pigmented Epithelial Detachment, *RPE* Retinal Pigmented Epithelium, *SD* Standard Deviation, *VRL* Vitreoretinal Lymphoma

## Discussion

Timely diagnosis of VRL is crucial due to the association with CNS lymphoma and increased mortality rates [13]. In addition, early detection ensures appropriate management and improves patient outcomes [14]. Unfortunately, since VRL signs and symptoms often resemble other ocular conditions, delayed diagnosis is common [7]. Therefore, the identification of characteristics strongly associated with VRL could improve the speed and accuracy of diagnosis. In this study, we systematically reviewed the SD-OCT features of VRL and compared these to non-infectious uveitides which had similar clinical presentations (intermediate uveitis and sarcoid posterior uveitis), to identify differentiating features that will improve diagnostic accuracy and facilitate prompt and appropriate intervention.

Previous studies have described OCT features of VRL. Barry et al. reviewed 32 eyes with VRL and noted the presence of hyper-reflective subretinal (53%) and intraretinal (18%) infiltrates, RPE undulation (15%), vitreous cells (15%), and sub-RPE deposits (9%) [15]. They concluded that hyperreflective subretinal infiltrates are unique to VRL. In a study of 6 eyes with retinitis-like VRL, Marchese et al. found that these cases had infiltrates in the subretinal and sub-RPE space (50%) and an increase in the retinal thickness (100%). Other noted features were retinal hyperreflective dots, hyporeflective cysts, and retinal “rounded roof” appearance that resembled OCT features of viral retinitis [16]. Similarly, Deak et al. found retinal deposits in 58.3% of cases (12 eyes) referred to as vertical hyperreflective columns, present between the nerve fiber layer and the RPE [11]. RPE irregularities were commonly detected in their patients [11]. Yang et al. studied 55 eyes with VRL and noted vitreous opacities (65%), preretinal deposits (13%), intraretinal deposits (15%), subretinal deposits (36%), RPE abnormalities (64%), and sub-RPE deposits (64%) [10].

OCT features have also been described for intermediate uveitis and sarcoid chorioretinitis. In sarcoidosis, findings include subretinal fibrosis and outer retinal infiltrations in the area of resolved granulomas, [17] intraretinal hyperreflective lesions, retinal thickening, subretinal and intraretinal fluid, and sub-RPE hyperreflective nodules [18, 19]. Retinal abnormalities are reported with intermediate uveitis. In addition to vitritis, 57% of patients with intermediate uveitis have ERM [20], and 8–26% have macular edema on OCT [21]. RPE loss/atrophy with hypertransmission defects into the choroid have also been reported in intermediate uveitis [22]. Many of these features overlap with those seen in VRL, making it challenging to distinguish between these entities without biopsy.

Only one previous study directly compared VRL and uveitis to determine the predictive value of specific OCT features [12]. The study included 45 eyes with VRL and 40 eyes with posterior uveitis (syphilitic uveitis, sympathetic ophthalmia, and multifocal choroiditis). Both VRL and uveitis had comparable rates of subretinal infiltrates (62% vs. 55%, *p* = 0.5), sub-RPE infiltrates (80% vs. 80%,* p* = 1), and RPE thickening (40% vs. 32.5%,* p* = 0.5). VRL eyes had significantly more frequent preretinal (45% vs. 7.5%, *p* < 0.001) and intraretinal (44% vs. 20%,* p* < 0.05) infiltrates and lower rates of choroidal infiltrates (23% vs. 97.5%, *p*< 0.001). Specific OCT findings that were only observed in eyes with VRL included complete or mixed vertical hyperreflective lesions, diffuse intraretinal hyperreflective lesions, and confluent RPE detachments. The uveitides included in the study were selected based on their shared anatomical retinal involvement with VRL patients, and as a representation of infectious, non-infectious, and idiopathic uveitis. However, the clinical presentations of the uveitides typically have less overlap with VRL than the uveitides included in our study (intermediate uveitis and sarcoid posterior uveitis). Clinical suspicion for syphilitic uveitis is often suggested by the medical history and extraocular signs and symptoms, supported by clinical examination, and confirmed by serological testing [23]; sympathetic ophthalmia occurs after a traumatic or surgical ocular injury [24]; and multifocal choroiditis is diagnosed by characteristic fluorescein and indocyanine green angiography features of small punched-out chorioretinal lesions that sometimes cause choroidal neovascularization [25].

The present study compares a large cohort of 95 eyes with VRL to 87 eyes with intermediate uveitis or sarcoid posterior uveitis, two uveitides that have clinical features that are very similar to VRL. OCT features more frequently observed in VRL included preretinal deposits (31.6%), intraretinal infiltrates (34%), outer retinal atrophy (22.1%), subretinal deposits (39%), and RPE atrophy (8.4%). Several OCT findings were only present in eyes with VRL: inner retinal hyperreflective spots (15%), RPE changes (thickening 13.7%, rippling 16.8%, and irregularity 24.2%), and sub-RPE deposits (34.7%). A larger study with inclusion of a broad array of uveitides would be needed to determine if these features are specific to VRL alone, but such features may be helpful distinguishing characteristics when considering the differential diagnosis of VRL compared to intermediate or sarcoid posterior uveitis. In contrast, ERM, central macular thickening, macular edema, and subretinal fluid were more likely to occur in the eyes with uveitis, consistent with other studies of chronic uveitis [26]. After age- and sex-adjusted univariate and multivariate analysis, the absence of ERM and the absence of central macular thickening were the most predictive features of VRL as opposed to uveitis.

Based on univariate analysis, the features most predictive of VRL included preretinal deposits, intraretinal infiltrates, outer retinal atrophy, band and focal subretinal deposits, RPE changes, and the absence of ERM and central macular thickening. These findings help confirm the prior study findings that preretinal deposits, intraretinal infiltration, banded subretinal infiltration, and confluent RPE detachment are highly suggestive of VRL. In contrast, the current study found no meaningful association of vitreous debris alone, most likely because all three entities (VRL, intermediate uveitis, and sarcoid posterior uveitis) usually have vitreous cell and debris.

Consistent with previous studies [13, 27–29], in this series, bilateral involvement was less common in VRL (76.8%) compared to the cases of intermediate uveitis and posterior sarcoid uveitis (93.3%). In the literature, at initial presentation, VRL is bilateral in 40 to 70% of patients [3, NaN], compared to 70 to 90% of intermediate and posterior uveitis cases [29–31]. As expected, patients with VRL were older than with uveitis in our study.

Study limitations include those inherent to a retrospective study design such as potential observation bias. Due to the rarity of the diseases of interest, the sample size was small, which limited our ability to include multiple variables within a single multivariate regression model, potentially generating confounding variables. Data were extracted from a single institution with a predominantly white patient population. Future multicenter prospective studies should be conducted to validate our results. Finally, analysis of OCT features was limited to those that have been previously described, and detection of features was limited by the OCT cuts that were available. Features could have been missed in the retinal periphery if targeted OCT cuts were not obtained, and additional OCT features that have not been previously defined could have been missed. Applications of artificial intelligence and machine learning could ultimately disclose information not discernable to the human eye.

In conclusion, we investigated SD-OCT features in VRL compared to non-infectious uveitis. Features most predictive of VRL, based on univariate analysis, were the presence of intraretinal infiltrates, RPE changes, the absence of ERM, and central macular thickening. By applying multivariate analysis with age- and sex-adjustment, only absence of ERM and absence of central macular thickening remained significant. SD-OCT is a non-invasive and readily available imaging tool that can help raise suspicion for VRL diagnosis. Future larger studies are warranted to confirm and validate key OCT features to distinguish malignancy from inflammatory intraocular disease.

## Data Availability

All data generated and analyzed during this study are included in this article. Further inquiries concerning the data that support the findings of this study are available upon reasonable request which can be directed to the corresponding author

## Abbreviations

CME: Cystoid macular edema
CNS: Central nervous system
OCT: Optical coherence tomography
OR: Odds ratio
RPE: Retinal pigmented epithelium
SD-OCT: Spectral domain optical coherence tomography
VRL: Vitreoretinal lymphoma
